# Textbook outcome after major hepatectomy for perihilar cholangiocarcinoma — definitions and influencing factors

**DOI:** 10.1007/s00423-022-02467-y

**Published:** 2022-03-04

**Authors:** Christian Benzing, Lena Marie Haiden, Felix Krenzien, Alexa Mieg, Annika Wolfsberger, Cecilia Filiz Atik, Nora Nevermann, Uli Fehrenbach, Wenzel Schöning, Moritz Schmelzle, Johann Pratschke

**Affiliations:** 1 Department of Surgery, Campus Charité Mitte | Campus Virchow-Klinikum, Experimental Surgery and Regenerative Medicine, Charité – Universitätsmedizin Berlin, Augustenburger Platz 1, 13353 Berlin, Germany; 2 Department of Radiology, Charité - Universitätsmedizin Berlin, Berlin, Germany

**Keywords:** Textbook outcome, Perihilar cholangiocarcinoma, Major hepatectomy

## Abstract

**Purpose:**

The concept of “textbook outcome” (TO) as composite quality measure depicting the ideal surgical has not yet been defined for patients undergoing major hepatectomy (MH) for perihilar cholangiocarcinoma (PHC). This study sought to propose a uniform definition through a systematic literature review as well as to identify patient- or procedure-related factors influencing TO.

**Methods:**

In this retrospective study, we analyzed all patients undergoing MH for PHC at our department between January 2005 and August 2019. After conducting a systematic literature search, we defined TO as the absence of 90-day mortality and major complications, no hospital readmission within 90 days after discharge, and no prolonged hospital stay (<75. percentile). A binary logistic regression analysis was performed to identify factors influencing TO.

**Results:**

Of 283 patients, TO was achieved in 67 (24%) patients. Multivariate analysis revealed that preoperative biliary drainage was associated with a decreased (OR= 0.405, 95% CI: 0.194–0.845, *p*=0.016) and left-sided-resection (OR= 1.899, 95% CI: 1.048–3.440, *p*=0.035) with increased odds for TO. Overall survival (OS) and DFS (disease-free survival) did not differ significantly between the outcome groups (OS: *p*=0.280, DFS: *p*=0.735). However, there was a trend towards better overall survival, especially in the late course with TO.

**Conclusion:**

Our analysis proposed a uniform definition of TO after MH for PHC. We identified left hepatectomy as an independent factor positively influencing TO. In patients where both right- and left-sided resections are feasible, this underlines the importance of a careful selection of patients who are scheduled for right hepatectomy.

**Supplementary Information:**

The online version contains supplementary material available at 10.1007/s00423-022-02467-y.

## Introduction

Perihilar cholangiocarcinoma (PHC) is a rare malignant tumor arising from the bile duct that is characterized by a poor prognosis [1]. So far, major hepatectomy (MH) is the only established, potentially curative treatment for patients with PHC. Radical surgical approaches such as hilar en bloc resection described by Neuhaus and colleagues were able to increase overall survival rates in the last years [2, 3]. However, high postoperative morbidity and mortality still remain unsatisfactory, despite advances in preoperative workup such as portal vein embolization (PVE) and improvements in perioperative management [4–6]. Diagnosis at an advanced stage, which is often accompanied by bile duct obstruction, cholestasis, cholangitis, and poor liver function, and radical surgical approaches are associated with morbidity rates as high as 50–60% and substantial mortality rates ranging between 5 and 18%, even in high-volume centers [5, 7–11]. After resection, common complications are bile leakage, septic, or vascular complications; however, post-hepatectomy liver failure (PHLF) being associated with a high associated mortality is feared the most [7, 12].

Recently, the concept of the “textbook outcome” (TO) as composite quality measure depicting the ideal surgical outcome has been used especially in complex oncological surgery [13–16]. TO is achieved when certain predefined desirable outcomes are simultaneously present in a patient’s postoperative course representing a much more comprehensive summary of a patient’s hospitalization than one singular outcome parameter such as mortality [17]. There is no generally accepted definition of TO and different parameters are used to define TO depending on the type of surgery or tumor [18]. Traditional quality measures like postoperative mortality (e.g., 90-day mortality), postoperative morbidity, or length of stay (LOS) can often be found in the definition of TO, but histopathological factors such as the presence of an R0 resection are increasingly used as well [13, 19, 20]. As a comprehensive quality measure, TO can not only be used by patients and health care providers for assessing the quality of surgical care or hospital performance but also by surgeons to optimize preoperative workup and surgical care [19]. TO has not been established in PHC surgery yet and TO rates as well as potentially influenceable patient- and procedure-related factors TO are unknown. As it might help improve surgical management and thus postoperative morbidity and mortality, this study was conducted to define TO after major liver resection for PHC, identify prognostic factors predicting TO, and analyze the impact of achieving a TO on overall survival (OS) and disease-free survival (DFS).

## Methods

### Patients and study design

Patients undergoing MH in curative intent for PHC between January 2005 and August 2019 at the Department of Surgery, Campus Charité – Mitte and Campus Virchow Klinikum, Charité – Universitätsmedizin Berlin were retrospectively analyzed. This retrospective study was approved by the local ethics committee (EA2/006/16 and EA1/358/16). Variables included in the analysis were general patient characteristics such as gender, age, American Association of Anesthesiologists (ASA) score, and body mass index (BMI). Perioperative and histopathological data were recorded as well as data on overall survival (OS) and disease-free survival (DFS). Postoperative morbidity as classified by Dindo-Clavien during hospitalization, 30- and 90-day mortality, length of hospital stay, and intensive care unit stay, respectively, were recorded as well [21].

### Preoperative management

All patients who were referred to our institution for surgical treatment underwent a highly individualized and detailed workup. This routinely included computed tomography and/or magnetic resonance imaging of the chest and abdomen as well as endoscopic retrograde cholangiography (ERC). Biliary drainage with ERC and biliary stenting or percutaneous transhepatic cholangiodrainage (PTCD) was performed when necessary. Routinely, Carbohydrate antigen 19-9 (CA 19-9) was measured before resection. On patients with suspected peritoneal dissemination, diagnostic laparoscopy or laparotomy was performed.

### Surgical procedure

All patients who underwent MH for PHC were included in the analysis. The surgical resection was performed either as a right or left sided major hepatectomy with extrahepatic bile duct resection as described before [2, 3]. Biliary reconstruction was performed as end-to-side hepaticojejunostomy. Patients with extrahepatic bile duct resection alone or multivisceral resections, e.g., hepatoduodenopancreatectomy (HPD) were excluded from the analysis as well as patients with intrahepatic or distant metastases or local peritoneal carcinomatosis.

### Histopathology

In all cases, PHC was confirmed according to the histopathological reports of the resected specimen. Furthermore, data on resection and lymph node status as well as perineural sheath infiltration, microvascular infiltration, lymphangiosis carcinomatosa, and tumor differentiation were collected from pathology reports. Based on the collected data and the TNM classification valid at the time of resection, patients were assigned to the appropriate tumor stage according to the Union for International Cancer Control (UICC, 7th edition).

### Textbook outcome and postoperative course

As TO had not yet been defined for major liver resection for PHC, a systematic literature review was conducted to evaluate common TO definitions used in HPB surgery. TO was defined based on the results of this literature review and common complications after PHC resection mentioned in the established literature. The search terms “textbook outcome” and “textbook oncologic outcome” were shortened to “textbook outcom*” and “textbook oncologic outcom*” and furthermore combined with the Boolean operator AND the following search terms: “liver surgery,” “hepatectomy,” “resection of liver,” “pancreatic surgery,” “pancreaticoduodenectomy,” “resection of pancrea*,” “hepatopancreatic surgery.” The publication period was limited to the years 2010 to 2020. Only studies in English with TO as primary endpoint were considered. Study designs such as reviews or meta-analyses were excluded. In addition, studies that investigated hepatic resections performed laparoscopically were excluded. TO was defined as the absence of 90-day mortality and major complications (i.e., > grade II according to Dindo-Clavien), no hospital readmission within 90 days after discharge, and no prolonged hospital stay (i.e., <75. percentile). The dichotomous textbook outcome was achieved when all four abovementioned individual criteria were observed in one patient after resection. Patients that could not be classified as either TO or NTO due to missing data were excluded from the analysis.

### Follow-up

Patients were followed up in the outpatient clinic or with their general practitioner. Check-ups routinely included testing of CA 19-9 serum levels and abdominal ultrasound, CT, or MRI. Whether adjuvant chemotherapy was performed was recorded as well.

### Statistics

Statistical analysis was performed using IBM SPSS Statistics for Macintosh Version 25.0 (IBM Corp., Armonk, NY, USA). A *p*-value < 0.05 was considered statistically significant. Continuous parameters are presented as median and range and statistically compared with the non-parametric Mann-Whitney-*U*-test. Categorical data are displayed as counts and percentages and are compared using the chi-squared test or Fisher’s exact test, when necessary. To identify independent factors influencing TO, a binary logistic regression analysis was performed. Results are reported as odds ratio (OR) and 95% confidence interval (95% CI). Prognostic factors were included in the regression model when a significant influence on TO was detected in univariate analysis. The variables age and gender were included in multivariate analysis regardless of significance. Survival was estimated with the Kaplan-Meier method and compared between the outcome groups with the log-rank test. Patients who died within 90 days of surgery were not included in the survival analyses. A subgroup analysis was performed with all patients surviving at least 30 months.

## Results

### Definition of TO

**Fig. 1:**
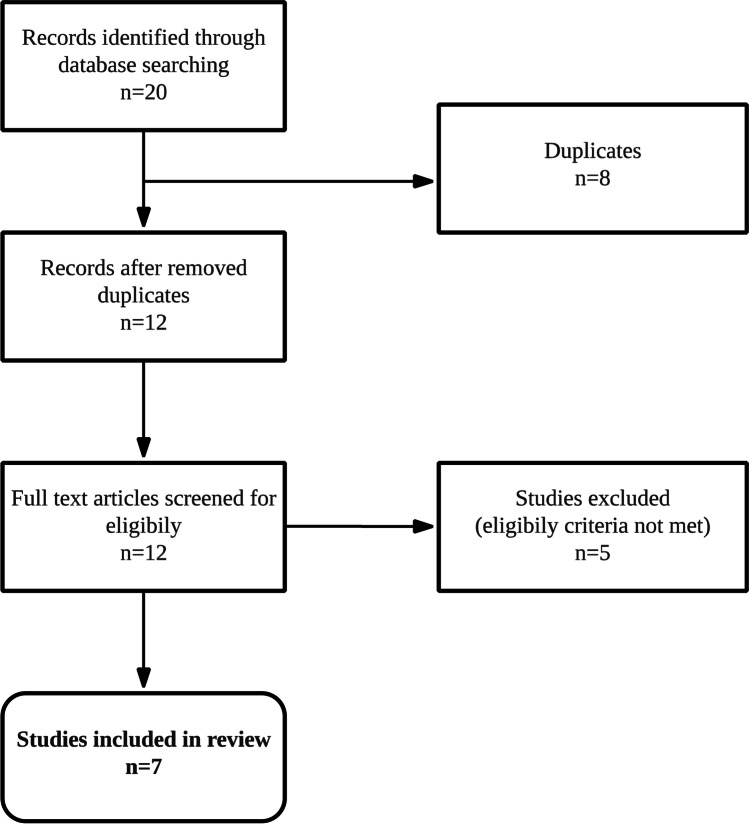
Flowchart of search algorithm

A total of 20 records were identified through database searching. After removal of duplicates, 12 records were screened for eligibility. After excluding sources that did not meet the inclusion criteria, a total of 7 studies in HPB surgery with TO as primary endpoint were identified for the final review. Figure 1 summarizes the search algorithm, whereas Table 1 and Table 2 give a summary on included studies. All studies were retrospective and multicenter studies that were published between 2019 and 2020. The smallest and largest series contained 687 and 21234 patients, respectively. Postoperative mortality, length of stay, and hospital readmission were part of the TO definition in all seven studies. Four records [17, 19, 20, 24] used 30-day mortality and 30-day readmission, whereas three studies [13, 22, 23] used a span of 90 days instead. Histopathological criteria were used in four studies [13, 17, 19, 20] to define TO. This included tumor-free resection margins in all four studies [13, 17, 19, 20] and absence of lymph node metastases in one [20] study. Adjuvant chemotherapy and the need for postoperative transfusion were part of the TO definition in two studies [17, 20]. One study was not able to get detailed information on postoperative morbidity and used LOS instead [20]. Specific complications such as bile leakage or postoperative pancreas fistula were part of the TO definition on one study [24]. All studies investigated TO rates for different tumor entities or HPB procedures, and most studies sought to identify TO-influencing factors after surgery (Table 2).

**Table 1:** TO studies included in review

Author	Year of publication	Study characteristics	Year of inclusion	Number of patients	TO definition used
Heidsma et al. [13]	2020	Retrospective, multicenter	2000–2016	821	LOS, RA90, 90DM, R0, PK<III
Mehta et al. [22]	2020	Retrospective, multicenter	2013–2017	21234	LOS, RA90, 90DM, PK0
Merath et al. [17]	2019	Retrospective, multicenter	1993–2015	687	LOS, 30DM, RA30, R0, PK0, Tr0
Merath et al. [23]	2020	Retrospective, multicenter	2013–2015	13467	LOS, RA90, 90DM, PK0
Sweigert et al. [20]	2020	Retrospective, multicenter	2006–2015	18608	LOS, RA30, 30DM, aCT, N0, R0
Tsilimigras et al. [19]	2020	Retrospective, multicenter	2005–2017	1829	LOS, RA30, 30DM, R0, PK<III
Van Roessel et al. [24]	2020	Retrospective, multicenter	2014–2017	3341	LOS, RA30, 30DM, BL, PK<III, PPF, PPH

*TO* textbook outcome, *RA30* no 30-day readmission, *LOS* no prolonged hospital stay, *30DM* no 30-day mortality, *RA90* no 90-day readmission, *90DM* no 90-day mortality, *PK0* no postoperative complications according Dindo-Clavien [21], *PK<III* no major postoperative complications according Dindo-Clavien (i.e., grade <III) [21], *R0* tumor-free resection margin after resection, *Tr0* no perioperative blood transfusion, *PPF* no postoperative pancreas fistula, *BL* no bile leakage, *PPH* no post-pancreatectomy hemorrhage, *aCT* adjuvant chemotherapy, *N0* absence of lymph node metastasis

**Table 2:** TO studies included in review

Author	Tumor entity	Procedure	Primary endpoint
Heidsma et al. [13]	Pancreatic neuroendocrine tumor	Pancreatoduodenectomy, distal pancreatectomy, enucleation	Incidence of TO, influence on DFS; TO and different surgical approaches
Mehta et al. [22]	Malignant tumor of pancreas or liver	Minor or major liver resection, minor or major pancreatic resection	Incidence of TO, TO rates according to hospital type
Merath et al. [17]	ICC	Hepatectomy	Incidence of TO, factors associated with TO, TO rates according to hospital type
Merath et al. [23]	All indications for mentioned procedure	Minor or major liver resection, minor or major pancreatic resection	Incidence of TO, factors associated with TO, TO and different surgical approaches
Sweigert et al. [20]	Pancreatic adenocarcinoma	Pancreatoduodenectomy	Incidence of TO, factors associated with TO, influence on OS
Tsilimigras et al. [19]	HCC, ICC	Hepatectomy	Incidence of TO, factors associated with TO, influence on OS
Van Roessel et al. [24]	All indications for mentioned procedure	Pancreatoduodenectomy, distal pancreatectomy	Incidence of TO, factors associated with TO, TO rates according to hospital type

*TO* textbook outcome, *DFS* disease-free survival, *HCC* hepatocellular cholangiocarcinoma, *ICC* intrahepatic cholangiocarcinoma, *OS* overall survival

### Patients’ characteristics

In this study, two hundred and eighty-three patients who underwent surgical resection for PHC in curative intent between January 2005 and August 2019 and met the inclusion criteria were analyzed. The cohort comprised 171 (60%) male and 112 (40%) female patients with a mean age of 65 (33–86) years. The majority of patients was grouped in either ASA 2 (56%, *n*=159) or ASA 3 (38%, *n*=107), thus suffering from pre-existing conditions. Table 3 summarizes all patient characteristics of the total cohort. The majority of patients presented with Bismuth Type IV PHC or advanced UICC stages (IIIB–IVA: 48%, *n*=134). Tumor-free resection margins were achieved in 68% (*n*=189) of all patients, whereas 48% (*n*=132) patients had histopathologically confirmed local lymph node metastases. Most patients presented with a moderate tumor differentiation (G2: 68%, *n*=190). A total of 114 patients (40%) were diagnosed with cholangitis preoperatively. Most patients underwent biliary drainage (86%, *n*=243) before surgery. Portal vein embolization was part of the preoperative workup in 46% (*n*=129) of patients, all receiving right-sided resections. The majority of patients underwent right-sided liver resection (63%, *n*=178) of which 58% (*n*=165) were right trisectionectomies. Thirty-seven percent (*n*=105) underwent left-sided liver resection, of which 23% (*n*=64) were left trisectionectomies. En bloc portal vein resection was performed in 57% (*n*=162) of cases.

**Table 3:** Patient characteristics

	Resected perihilar cholangiocarcinoma
	*n* = 283
Age^1^	65 (33–86)
BMI^1^	24.6 (16.0–38.3)
Gender (male)^2^	171 (60)
ASA score^2^	
1	13 (5)
2	159 (56)
3	107 (38)
4	4 (1)
Bismuth-Corlette^2^	
I	13 (5)
II	20 (7)
IIIa	72 (26)
IIIb	55 (20)
IV	116 (42)
UICC stage^2^	
I	12 (4)
II	100 (36)
IIIa	32 (12)
IIIb	127 (46)
IVa	7 (2)
IVb	0 (0)
Resection margin^2^	
R0	189 (68)
R1	89 (32)
Lymph node status^2^	
N0	146 (53)
N+	132 (48)
Microvascular invasion^2^	
Yes	48 (19)
No	206 (81)
Histopathological grading^2^	
Grade 1	15 (5)
Grade 2	190 (68)
Grade 3	73 (26)
Perineural sheath infiltration^2^	
Yes	207 (88)
No	28 (12)
Lymphangitis carcinomatosa^2^	
Yes	100 (41)
No	147 (59)
T stage^2^	
is	1 (0)
1	19 (7)
2a	81 (29)
2b	94 (33)
3	81 (29)
4	7 (3)
Preoperative biliary drainage^2^	
Yes	243 (86)
No	40 (14)
Portal vein embolization^2^	
Yes	129 (46)
No	154 (54)
Preoperative cholangitis^2^	
Yes	114 (40)
No	169 (60)
Resection side^2^	
Left hepatectomy	105 (37)
Extended left hepatectomy	41 (15)
Left trisectionectomy	64 (23)
Right hepatectomy	178 (63)
Extended right hepatectomy	13 (5)
Right trisectionectomy	165 (58)
Portal vein resection^2^	
Yes	162 (57)
No	121 (43)
Operating time (min)	385 (112–849)
Complications (Clavien-Dindo)^2^	
None	29 (10)
I	12 (4)
II	56 (20)
IIIa	84 (30)
IIIb	51 (18)
IVa	6 (2)
IVb	1 (0)
V	44 (16)
Severe complications (grade IIIa–V)	186 (66)
Preoperative ALAT (U/l)	66 (9–1270)
Preoperative ASAT(U/l)	55 (13–3352)
CA 19-9 (kU/l)^1^	79 (1–32670)
ICU stay (days)^1^	4 (1–123)
Hospital stay (days)^1^	23 (3–213)
30-day mortality^2^	22 (8)
90-day mortality^2^	42 (15)
Hospital readmission	61 (22)
Prolonged hospital stay	72 (25)
Preoperative chemotherapy	
Yes	10 (4)
No	273 (96)
Adjuvant chemotherapy	
Yes	64 (23)
No	209 (77)

^1^Data is presented as median and range; ^2^Data is presented as count and proportions (%)

### Postoperative morbidity, mortality, and textbook outcome

Most patients suffered from postoperative complications (90%, *n*=254), while major complications (grade IIIa–V) occurred in 66% (*n*=186) [21]. Common complications were infection (53%, *n*=150), pleural effusion (34%, *n*=96), bile leakage (30%, *n*=86), and postoperative liver failure (26%, *n*=74). Supplementary Table S1 gives an overview of the specific postoperative complications. After resection, the median length of hospital stay was 23 (3–213) days. The 30-day and 90-day mortality were 8% (*n*=22) and 15% (*n*=42). TO could be achieved in 24% (*n*=67), severe postoperative complications where the main reason not to be included in the TO group. From all patients, 22% (*n*=61) had to be readmitted or had a prolonged hospital stay (25%, *n*=72), thus not meeting the TO criteria. Some differences in patients’ characteristics were noted among patients who achieved TO versus patients who did not (Table 4). Patients with TO showed lower preoperative CA 19-9 levels (53 kU/l vs. 95 kU/l, *p*=0.047) and did less frequently undergo preoperative biliary drainage (73% vs. 90%, *p*=0.001). Left-sided hepatectomy was also associated with a higher TO rate when compared to right hepatectomy (52% vs. 32%, *p*=0.003). With regard to right-sided resections only side and extent of resection, TO is achieved in 18% (*n*=29) of right trisectionectomies versus 23% (*n*=3) after standard major hepatectomy (*p*=0.619). For left-sided resections, TO is achieved in 33% (*n*=21) after left trisectionectomy vs. 34% after standard major hepatectomy (*n*=14, *p*=0.888).

**Table 4:** Patient characteristics according to outcome group (TO versus NTO)

	TO	NTO	*p* value
	*n* = 67	*n* = 216	
Age^1^	64 (38–81)	65 (33–86)	0.696
BMI^1^	24.6 (16.0–35.0)	24.9 (16.0–38.3)	0.417
Gender (male)^2^	37 (55)	134 (62)	0.319
ASA score^2^			0.927
1	4 (6)	9 (4)	
2	38 (57)	121 (56)	
3	24 (36)	83 (38)	
4	1 (2)	3 (1)	
Bismuth-Corlette^2^			0.326
I	3 (5)	10 (5)	
II	2 (3)	18 (9)	
IIIa	14 (21)	58 (28)	
IIIb	17 (26)	38 (18)	
IV	30 (46)	86 (41)	
UICC stage^2^			0.489
I	4 (6)	8 (4)	
II	27 (40)	73 (35)	
IIIa	6 (9)	26 (12)	
IIIb	27 (40)	100 (47)	
IVa	3 (5)	4 (2)	
Resection margin^2^			0.892
R0	46 (69)	143 (68)	
R1	21 (31)	68 (32)	
Lymph node status^2^			0.284
N0	39 (58)	107 (51)	
N+	28 (42)	104 (49)	
Microvascular invasion^2^			0.378
Yes	9 (15)	39 (20)	
No	51 (85)	155 (80)	
Histopathological grading^2^			0.128
Grade 1	4 (6)	11 (5)	
Grade 2	51 (77)	139 (66)	
Grade 3	11 (17)	62 (29)	
Perineural sheath infiltration^2^			0.347
Yes	43 (84)	164 (89)	
No	8 (16)	20 (11)	
Lymphangitis carcinomatosa^2^			0.094
Yes	18 (31)	82 (57)	
No	40 (69)	107 (43)	
T stage^2^			0.399
is	0 (0)	1 (0)	
1	6 (9)	13 (6)	
2a	18 (27)	63 (29)	
2b	26 (39)	68 (32)	
3	14 (21)	67 (31)	
4	3 (5)	4 (2)	
Preoperative biliary drainage			0.001
Yes	49 (73)	194 (90)	
No	18 (27)	22 (10)	
Portal vein embolization			0.016
Yes	22 (33)	107 (50)	
No	45 (67)	109 (50)	
Preoperative cholangitis			0.571
Yes	25 (37)	89 (41)	
No	42 (63)	127 (59)	
Resection side^2^			0.003
Left hepatectomy	35 (52)	70 (32)	
Extended left hepatectomy	14 (21)	27 (13)	
Left trisectionectomy	21 (31)	43 (20)	
Right hepatectomy	32 (49)	146 (68)	
Extended right hepatectomy	3 (5)	10 (5)	
Right trisectionectomy	29 (43)	136 (63)	
Portal vein resection^2^			0.018
Yes	30 (45)	132 (61)	
No	37 (55)	84 (39)	
Operating time (min)	375 (232–547)	391 (112–849)	0.170
Severe complications (grade IIIa–V)	0 (0)	186 (86)	<0.001
Preoperative ALAT (U/l)^1^	68 (14–482)	66 (9–1270)	0.446
Preoperative ASAT (U/l)^1^	65 (17–430)	54 (13–3352)	0.919
CA 19-9 (kU/l)^1^	53 (1–32670)	95 (1–23049)	0.047
ICU stay (days)^1^	2 (2–18)	5 (1–123)	<0.001
Hospital stay (days)^1^	16 (7–37)	29 (3–213)	<0.001
Preoperative chemotherapy			0.300
Yes	1 (2)	9 (4)	
No	66 (98)	207 (96)	
Adjuvant chemotherapy			0.237
Yes	18 (29)	46 (22)	
No	44 (71)	165 (78)	

^1^Data is presented as median and range; ^2^Data is presented as count and proportions (%)

### Binary logistic regression analysis of factors influencing textbook outcome

To detect independent factors influencing TO, variables shown in Table 5 that showed significant influence on TO in univariable analysis were included in a binary regression analysis model (Table 5). The regression analysis identified preoperative biliary drainage (OR= 0.405, 95% CI: 0.194–0.845, *p*=0.016) and left-sided-resection (OR= 1.899, 95% CI: 1.048–3.440, *p*=0.035), as independent factors influencing TO, whereas left-sided resection was associated with higher and preoperative biliary drainage with lower odds of TO. Despite significant differences in univariable analysis, tumor differentiation was not an independent predictor for TO (OR= 0.547, 95% CI: 0.263–1.137, *p*=0.106). The patient-related factors age, sex, and ASA score had no impact on TO (age: *p*=0.392, sex: *p*=0.456, ASA: *p*=0.714).

**Table 5:** Univariable and multivariable analysis of factors influencing textbook outcome in all resected patients

All patients (*n*=283)				
Variable	Univariable		Multivariable	
	OR (95% CI)	*p* value	OR (95% CI)	*p* value
Age	0.993 (0.968–1.018)	0.563	0.989 (0.963–1.015)	0.392
Gender (male)	0.755 (0.434–1.314)	0.320	0.800 (0.444–1.440)	0.456
Body mass index (kg/m^2^)	0.960 (0.892–1.034)	0.282		
ASA (>2)	0.900 (0.511–1.583)	0.714		
Preoperative ALAT (U/l)	1.000 (0.998–1.002)	0.825		
Preoperative ASAT (U/l)	0.999 (0.997–1.001)	0.457		
T stage (>2b)	0.694 (0.374–1.290)	0.248		
UICC (>IIIA)	0.834 (0.480–1.449)	0.520		
Preoperative CA 19-9 (kU/l)	1.000 (1.000–1.000)	0.999		
Preoperative drainage (yes)	0.309 (0.154–0.620)	0.001	0.405 (0.194–0.845)	0.016
Resection side (left-sided resection)	2.281 (1.306–3.984)	0.004	1.899 (1.048–3.440)	0.035
Preoperative cholangitis (yes)	0.849 (0.483–1.494)	0.571		
Histopathological grading (>G2)	0.484 (0.237–0.986)	0.046	0.547 (0.263–1.137)	0.106
Perineural sheath infiltration (Pn1)	0.655 (0.270–1.590)	0.350		
Lymphovascular invasion (L1)	0.587 (0.314–1.098)	0.096		
Microvascular invasion (V1)	0.701 (0.318–1.547)	0.379		
Lymph node status (N+)	0.739 (0.424–1.287)	0.285		

### Survival analyses

**Fig. 2:**
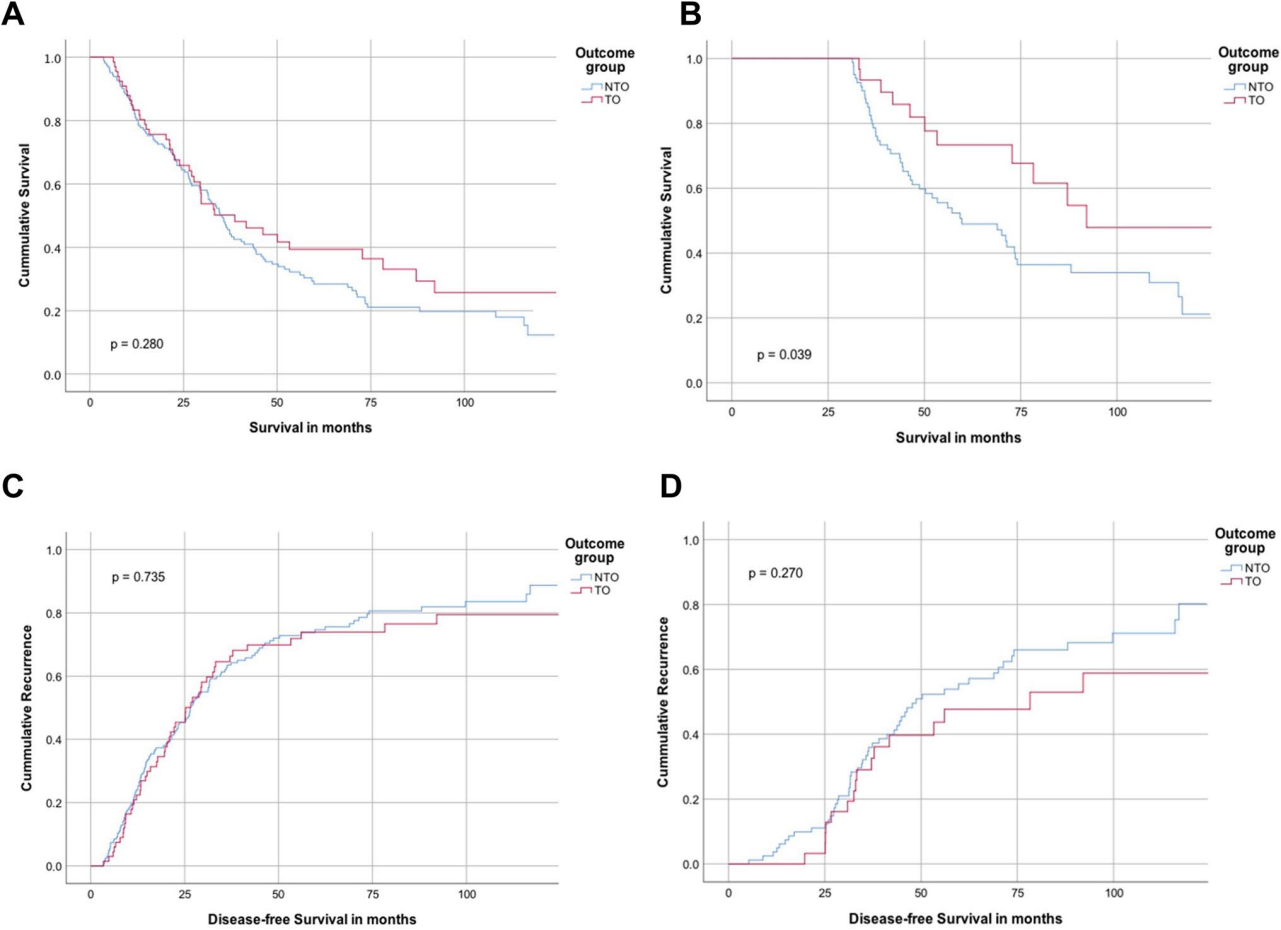
Overall survival and disease-free survival according to outcome group. Kaplan-Meier curves of (**A**) overall survival of all resected patients with perihilar cholangiocarcinoma excluding 90-day mortality according to outcome group; (**B**) overall survival of all resected patients surviving >30 months according to outcome group; (**C**) disease-free survival of all resected patients with perihilar cholangiocarcinoma excluding 90-day mortality according to outcome group; and (**D**) disease-free survival of all resected patients with perihilar cholangiocarcinoma surviving >30 months according to outcome group

Median OS of all patients was 29 (24–35) months after resection, whereas DFS was 22 (17–26) months. After excluding patients who died within 90 days after resection, no significant difference between the two groups in either OS (*p*=0.280) or DFS (*p*=0.735) analysis could be detected. However, a trend towards better overall survival in patients with TO especially in the late course could be shown that failed to reach statistical significance. Subgroup analyses of patients surviving at least 30 months (Supplementary Table S2) after resection showed better overall survival in patients with TO compared to patients without TO (92 versus 60, *p*=0.039). There was no difference in DFS between patients surviving at least 30 months (*p*=0.270, Figure 2A–D).

## Discussion

In recent years, the concept of the TO as a quality measure depicting the ideal surgical outcome has been used in complex oncological surgery, especially in the field of HBP surgery. A recently published multi-center study by Mueller et al. has examined the outcome after PHC surgery for so-called benchmark cases [25]. This study marks an important milestone in the field of PHC surgery since it provides a definition of what is to be considered a benchmark case. However, this is the first study dealing with TO definitions in PHC patients undergoing major hepatectomy. We were able to identify relevant studies dealing with TO in the field of HBP surgery and proposed a TO definition for PHC patients. Furthermore, we were able to identify left hepatectomy as a factor that was independently associated with TO.

TO was defined as the absence of 90-day mortality and major complications (i.e., > grade II according to Dindo-Clavien), no hospital readmission within 90 days after discharge, and no prolonged hospital stay (i.e., <75. percentile). According to the literature review, the exclusion criteria for TO were 90-day mortality, severe complications (≥IIIa according to Dindo-Clavien), unplanned readmission, and prolonged hospital stay [21]. When it comes to the definition of postoperative mortality, studies differ greatly, as some used 30-day mortality, others 90-day mortality [13, 17, 22, 23, 26]. Defining postoperative mortality as 30-day mortality may lead to an underestimation of the actual perioperative mortality after liver resection by up to 50%. After an initial steep increase in postoperative mortality, a more or less constant mortality rate is observed after 90 days [5, 27]. Indicators of postoperative morbidity reported in the analyzed TO studies were length of hospital stay (LOS), grade of complications, or specific complications. There is a known high correlation between LOS and incidence of complications [18].

Nevertheless, there is a potential bias when only reporting LOS [18, 20]. LOS may not necessarily represent a surgical quality measure, as it may be prolonged by external factors such as inadequately ensured home care at discharge and not morbidity-related [18] or cultural aspects [5, 17]. For these reasons, a second morbidity measure was added to the “prolonged hospital stay” parameter in the present work. Specific complications to define TO as used by van Roessel et al. were not used because patients often develop more than one complication. Instead of using “no morbidity” as a measure [23], major complications (≥IIIa according to Dindo-Clavien) were chosen as a parameter in this analysis, because major complications are frequently observed after MH for PHC [5, 21–23, 26]. Although R0 status was part of the TO definition in half of the TO studies [13, 17, 19, 20], we decided not to include it in the definition of TO in PHC surgery for several reasons. First, the perihilar region is narrow and R1 resection is not uncommon in PHC surgery [28–30] and patients benefit from surgical resection even in case R1 status is obtained. Second, especially in the subset of lymph node-positive patients which counts up to almost 50%, survival is independent of R status [30]. Third, PHC surgery is characterized by high perioperative morbidity and mortality. Thus, TO should rather be defined by an uneventful postoperative course than histopathological criteria.

Overall, a TO rate of 24% (*n*=67) was observed in the present study. This is consistent with TO rates obtained after surgical procedures in the field of HBP surgery [17, 19, 20]. Opposite to the findings in other studies [17, 19], age was not found to be influencing TO in the present study. Poor histologic differentiation had a negative impact on the development of a TO in the present study. In general, poor grading is considered a risk for early tumor recurrence and decreased OS [3, 31–33]. Additionally, however, these factors may also reflect an aggressive tumor biology indicating advanced tumor disease. This is related to frailty, which leads to increased morbidity and mortality [34].

In the present work, left hepatectomy could be identified as an independent factor favoring TO which is in line with another study [19] compared with right hepatectomy. This is due to a significantly smaller future liver remnant (FLR), which is a risk factor for PHLF which consecutively leads to increased postoperative mortality rates [7, 35]

As a second independent factor influencing TO, we found that preoperative biliary drainage to be associated with not achieving TO. Similar to the results of this work, Zhang et al. showed a significantly increased morbidity rate in patients with biliary drainage [36]. This is likely attributable to advanced disease as well as preoperative cholangitis; thus, drainage should be considered a surrogate parameter for advanced disease.

The median overall survival in the current study in the overall cohort was 29 (24–35) months, which is in line with other studies (13–40 months) [10, 28, 29, 37, 38]. Some previous studies in the field of HBP surgery have shown improved OS and DFS if TO is achieved [13, 19, 20, 39]. There was no significant difference in survival between the TO and NTO groups. However, in the long-term (beginning from month 30), there was a clear trend towards a better OS and DFS in the TO group compared to the NTO group. Reasons for improved long-term survival when TO is achieved may be a timely connection to adjuvant chemotherapy. This can be delayed or not occur at all in patients with major complications. In this work, a higher number of patients in the TO group tended to receive adjuvant chemotherapy in the overall cohort which can have a positive effect on OS and DFS [40]. However, it is difficult to draw final conclusions since exact follow-up data including the applied chemotherapy regimen, dose, and information on, e.g., premature termination of adjuvant chemotherapy due to side effects are missing in a relevant proportion of patients. In general, recommendations for adjuvant chemotherapy included either gemcitabine (± cisplatin) or fluorouracil/capecitabine.

There are several limitations in the present study. First, it is a retrospective analysis which can lead to bias. Second, clear definitions of TO in PHC lack, so the comparability is limited. However, this is the first study proposing a definition of TO in patients with PHC undergoing MH based on a thorough literature review, which is evaluated and tested on a large cohort of patients.

## Conclusions

TO is a quality indicator that is not exclusively limited to reporting mortality, but more comprehensively represents a desired postoperative course. In the present study, we introduced the composite quality measure TO into PHC. This is of great importance in order to allow a comparison between studies or centers. Therefore, there is a great need for a uniform and robust TO definition in future studies. We identified left hepatectomy as an independent factor positively influencing TO. In cases with central tumors, where both right- and left-sided resections are feasible, this underlines the importance of a careful selection of patients who are scheduled for right hepatectomy, which are superior in terms of oncological radicality but associated with higher postoperative morbidity and mortality.

## Supplementary Information

Below is the link to the electronic supplementary material.

Supplementary file1 (DOCX 31 kb)
