# Supplementary material for: Textbook outcome after major hepatectomy for perihilar cholangiocarcinoma — definitions and influencing factors
Source: Langenbecks Arch Surg. 2022 Mar 4;407(4):1561–73. doi: 10.1007/s00423-022-02467-y (PMC9283152; doi:10.1007/s00423-022-02467-y)
Supplement: Supplementary file 1 — Supplementary file1 (DOCX 31 kb) [file 423_2022_2467_MOESM1_ESM.docx]

**Supplementary Table S1.** Specific complications after major hepatectomy for perihilar cholangiocarcinoma

|  | Resected perihilar cholangiocarcinoma |
| --- | --- |
|  | n = 283 |
| Anastomotic leakage ^2^ |  |
| Yes | 35 (12) |
| No | 248 (88) |
| Bile leakage ^2^ |  |
| Yes | 86 (30) |
| No | 197 (70) |
| Cholangitis^2^ |  |
| Yes | 51 (18) |
| No | 232 (82) |
| Intraabdominal abscess^2^ |  |
| Yes | 26 (9) |
| No | 257 (91) |
| Cardial complications ^2^ |  |
| Yes | 30 (11) |
| No | 253 (89) |
| Postoperative liver failure ^2^ |  |
| Yes | 74 (26) |
| No | 89 (74) |
| Postoperative hemorrhage^2^ |  |
| Yes | 32 (11) |
| No | 251 (89) |
| Kidney failure ^2^ |  |
| Yes | 55 (19) |
| No | 228 (81) |
| Pleural effusion ^2^ |  |
| Yes | 96 (34) |
| No | 187 (66) |
| Pneumonia ^2^ |  |
| Yes | 39 (14) |
| No | 244 (86) |
| Portal vein thrombosis ^2^ |  |
| Yes | 24 (9) |
| No | 259 (91) |
| Infection ^2^ |  |
| Yes | 150 (53) |
| No | 133 (47) |

^1 Data is presented as median and range, 2 Data is presented as count and proportions (%),^

**Supplementary Table S2.** Patient characteristics according to outcome group (TO versus NTO) in patients surviving >30months

|  | TO | NTO | *P* value |
| --- | --- | --- | --- |
|  | n = 31 | n = 81 |  |
| Age ^1^ | 64 (38-81) | 62 (34-82) | 0.623 |
| BMI ^1^ | 24.0 (20.0-31.2) | 24.3 (17.6-34.3) | 0.636 |
| Gender (male) ^2^ | 15 (48) | 51 (63) | 0.161 |
| ASA score ^2^ |  |  | 0.894 |
| 1 | 4 (13) | 8 (10) |  |
| 2 | 16 (52) | 44 (54) |  |
| 3 | 11 (35) | 29 (36) |  |
| 4 | 0 (0) | 0 (0) |  |
| Bismuth-Corlette ^2^ |  |  | 0.260 |
| I | 1 (3) | 5 (6) |  |
| II | 1 (3) | 6 (8) |  |
| IIIa | 5 (17) | 23 (30) |  |
| IIIb | 10 (33) | 13 (17) |  |
| IV | 13 (43) | 31 (40) |  |
| UICC Stage ^2^ |  |  | 0.446 |
| I | 4 (13) | 7 (9) |  |
| II | 19 (61) | 37 (46) |  |
| IIIa | 3 (10) | 11 (14) |  |
| IIIb | 5 (16) | 24 (30) |  |
| IVa | 0 (0) | 1 (1) |  |
| Resection margin ^2^ |  |  | 0.903 |
| R0 | 24 (77) | 62 (79) |  |
| R1 | 7 (23) | 17 (21) |  |
| Lymph node status ^2^ |  |  | 0.108 |
| N0 | 26 (84) | 55 (69) |  |
| N+ | 5 (16) | 25 (31) |  |
| Microvascular invasion ^2^ |  |  | 0.279 |
| Yes | 1 (4) | 7 (10) |  |
| No | 27 (96) | 61 (90) |  |
| Histopathological grading ^2^ |  |  | 0.318 |
| Grade 1 | 3 (10) | 9 (11) |  |
| Grade 2 | 23 (77) | 50 (63) |  |
| Grade 3 | 4 (13) | 21 (26) |  |
| Perineural sheath infiltration ^2^ |  |  | 0.608 |
| Yes | 17 (85) | 58 (89) |  |
| No | 3 (15) | 7 (11) |  |
| Lymphangitis carcinomatosa ^2^ |  |  | 0.077 |
| Yes | 5 (19) | 25 (37) |  |
| No | 22 (82) | 42 (63) |  |
| T Stage ^2^ |  |  | 0.124 |
| is | 0 (0) | 0 (0) |  |
| 1 | 4 (13) | 8 (10) |  |
| 2a | 8 (26) | 32 (40) |  |
| 2b | 15 (48) | 20 (25) |  |
| 3 | 4 (13) | 20 (25) |  |
| 4 | 0 (0) | 1 (1) |  |
| Preoperative biliary drainage |  |  | 0.008 |
| Yes | 21 (68) | 72 (89) |  |
| No | 10 (32) | 9 (11) |  |
| Portal Vein Embolization |  |  | 0.109 |
| Yes | 9 (29) | 37 (46) |  |
| No | 22 (71) | 44 (54) |  |
| Preoperative cholangitis |  |  | 0.469 |
| Yes | 13 (42) | 28 (35) |  |
| No | 18 (58) | 53 (65) |  |
| Resection side ^2^ |  |  | 0.033 |
| Left hepatectomy | 18 (58) | 29 (36) |  |
| Extended left hepatectomy | 6 (19) | 8 (19) |  |
| Left trisectionectomy | 12 (39) | 21 (26) |  |
| Right hepatectomy | 13 (42) | 52 (64) |  |
| Extended right hepatectomy | 0 (0) | 2 (3) |  |
| Right trisectionectomy | 13 (42) | 50 (62) |  |
| Portal vein resection ^2^ |  |  | 0.051 |
| Yes | 12 (39) | 48 (59) |  |
| No | 19 (61) | 33 (41) |  |
| Operating time (min) | 365 (232-547) | 380 (253-849) | 0.576 |
| Severe complications (grade IIIa–V) | 0 (0) | 65 (80) | <0.001 |
| Preoperative ALAT (U/l)^1^ | 91 (14-482) | 74 (11-910) | 0.547 |
| Preoperative ASAT (U/l)^1^ | 66 (17-392) | 54 (18-605) | 0.893 |
| CA 19-9 (kU/l) ^1^ | 65 (8-32670) | 54 (1-4150) | 0.934 |
| ICU stay (days) ^1^ | 2 (2-9) | 4 (1-70) | 0.031 |
| Hospital stay (days) ^1^ | 16 (9-32) | 30 (10-213) | <0.001 |
| Adjuvant Chemotherapy |  |  | 0.508 |
| Yes | 8 (28) | 17 (22) |  |
| No | 21 (72) | 62 (78) |  |

^1 Data is presented as median and range, 2 Data is presented as count and proportions (%)^
